# Cultural barriers to effective communication between Indigenous communities and health care providers in Northern Argentina: an anthropological contribution to Chagas disease prevention and control

**DOI:** 10.1186/1475-9276-13-6

**Published:** 2014-01-29

**Authors:** Ana Dell’Arciprete, José Braunstein, Cecilia Touris, Graciela Dinardi, Ignacio Llovet, Sergio Sosa-Estani

**Affiliations:** 1 Instituto de Efectividad Clínica y Sanitaria, Buenos Aires, Argentina; 2 Consejo Nacional de Investigaciones Científicas y Técnicas, Buenos Aires, Argentina; 3 Centro Nacional de Diagnóstico e Investigación de Endemo-epidemias (CeNDIE) / Instituto Nacional de Parasitología “Dr. Mario FatalaChaben” / Administración Nacional de Laboratorios e Institutos de Salud (ANLIS) “Dr. Carlos G. Malbrán”, Buenos Aires, Argentina; 4 Departamento de Posgrado, Universidad Nacional de Tres de Febrero, Buenos Aires, Argentina; 5 Departamento de Ciencias Sociales, Universidad Nacional de Luján, Buenos Aires, Argentina

**Keywords:** Aboriginal health, Chagas disease, Wichi, Pilaga, Argentina, Communication, Local health system

## Abstract

**Introduction:**

Ninety percent of the aboriginal communities of Argentina are located in areas of endemic vectorial transmission of Chagas disease. Control activities in these communities have not been effective. The goal of this research was to explore the role played by beliefs, habits, and practices of Pilaga and Wichi indigenous communities in their interaction with the local health system in the province of Formosa. This article contributes to the understanding of the cultural barriers that affect the communication process between indigenous peoples and their health care providers.

**Methods:**

Twenty-nine open ended interviews were carried out with members of four indigenous communities (Pilaga and Wichi) located in central Formosa. These interviews were used to describe and compare these communities’ approach to health and disease as they pertain to Chagas as well as their perceptions of Western medicine and its incarnation in local health practice.

**Results:**

Five key findings are presented: 1) members of these communities tend to see disease as caused by other people or by the person’s violation of taboos instead of as a biological process; 2) while the Pilaga are more inclined to accept Western medicine, the Wichi often favour the indigenous approach to health care over the Western approach; 3) members of these communities do not associate the vector with the transmission of the disease and they have little awareness of the need for vector control activities; 4) indigenous individuals who undergo diagnostic tests and accept treatment often do so without full information and knowledge; 5) the clinical encounter is rife with conflict between the expectations of health care providers and those of members of these communities.

**Conclusion:**

Our analysis suggests that there is a need to consider the role of the cultural patterning of health and disease when developing interventions to prevent and control Chagas disease among indigenous communities in Northern Argentina. This is especially important when communicating with these communities about prevention and control. These research findings might also be of value to national and provincial agencies in charge of decreasing the rates of Chagas disease among indigenous populations.

## Introduction

Chagas disease is an anthropozoonosis due to the parasite *Trypanosoma cruzi*. The parasite moves among humans, other mammals, and insectstriatomines, *T. infestans*, usually known as *vinchuca* in local Spanish [1]. The disease affects 10 million people in Latin America, killing 10,000 people per year [2,3]. Chagas disease transmission continues to be an important public health problem in Argentina. In 2009, the seroprevalence of *T. cruzi* infection was 4.2% in pregnant women and 1.5% in children under 14 years of age (Programa Nacional de Chagas). While there have been improvements in entomological surveillance and control of *T. infestans*, acceptable goals are still a long way off. In some regions of Argentina, namely the northeast, where there is a lack of systematic vector control, and vulnerable population groups like the indigenous people, there remains a high risk for disease transmission. The majority of the indigenous population lives in rural areas where the risk of vectorial transmission is present [4]. In these areas, rates of disease are much higher than national averages [5,6].

Activities implemented by the agencies responsible for the prevention and control of *Trypanosoma cruzi* transmission - spraying insecticides and entomological surveillance for vectorial transmission, prenatal care for congenital transmission, blood tests for transfusional transmission [7]- have been effective in the majority of non-indigenous communities. Nevertheless, among indigenous groups these control efforts have not had the same impact. This differential health outcome implies an inequity, i.e. a “systematic and remediable difference among population groups” [8], that has not been taken into account by the health policy. Although the health gap is well-known among policy-makers and experts, the guidelines laid out in the Plan 2011–2016 for the Control of Chagas Disease [9] do not specifically include measures aimed at addressing Aboriginal populations. National and provincial vector-control programs for *Trypanosoma cruzi* apply the same strategies in their interactions with all population groups, whether they be Creoles or Aboriginals of various ethnicities. In local health systems, the limited effectiveness of efforts has been interpreted as resulting from communication barriers faced by health professionals from Western and urban backgrounds in their treatment of indigenous patients. Accordingly, one example of the primary health care response has been to recruit young men and women from Aboriginal communities who have good command of Spanish to work as community health workers. In light of the results, this strategy should be considered insufficient. Linguistic differences are part of what makes this relationship difficult. However, there are also deep and often disregarded differences in the concepts of health, disease, and therapy, as well as etiquette [10].

Communication difficulties also have a significant impact on the quality of surveillance activities. The lack of timely reporting of re-infestation opens the way for renewed transmission, diminishes the effectiveness of treatment, and increases the risk of generating resistant strains of *T. cruzi*.

In our view, the emphasis placed by local health services on one-directional and instrumental communication takes for granted that: a) the health threat is clearly identified by the population at-risk, b) those considered health experts agree on the adopted course of action, c) the at-risk or currently affected population shares a similar degree of alarm to the extent that the message is clear [11]. In order for these assumptions to be met, notions and practices of health and disease on both sides of the communication relationship (i.e., the health system/indigenous population) must be aligned.

Consequently, a relevant research question is whether the information provided by the local health system allows for an understanding of the causes, prevention, and treatment of Chagas disease in an Aboriginal cultural framework. In order to respond to this question, in 2006 we undertook an empirical research of the interaction between the indigenous population and the health system in the Formosa Province. In this geographic area the endemic status of Chagas disease is exhibited by higher numbers of domestic triatomids infestation (33.2%), a prevalence of infection in the general population of 17.8% and of 8.6% in children under five [5].

The study looked at two indigenous groups, Pilaga and Wichi, which have different cultural and epidemiological characteristics. The Pilaga have an oral language (*qoml’aqtak*) from the Guaycurú language group and a variable command of Spanish, spoken by some adult men and youths of both sexes. Those who speak Spanish are semi-literate and use it only to communicate with other ethnic groups. In turn, the Wichi language (*Wichilhomtés*) belongs to the Mataco-Maká language group. As with the Pilaga, it is the only language used within their communities, although the proportion of monolingual speakers is higher than among the Pilaga. Wichi and Pilaga have experienced a strong influence of evangelism, although at different times; Pilaga came into contact with the Pentecostal Church in 1940s while Wichi adopted evangelic practices later, in the 80s [12]. This religious influence affects their everyday life. Both indigenous ethnic groups share rates of infestation and infection higher than those observed in the general population. However, the Wichi population has a rate of infection three times higher than the Pilaga population. The Pilaga’s strategies differs from those of the Wichi in that the former incorporated Western hygienic practices of a sedentary lifestyle while the Wichis’ resistance to change, in order to preserve their culture, makes harder their adaptation to it [13]. In addition to this, while Christianization introduced new syncretic forms of shamanism, but indigenous representations of the body, general wellness, causes of disease, and curative practices in general remain. Both indigenous communities share belief systems that differ from the Western notions of therapy.

Following the mandate of the provincial legal framework, community health workers with indigenous background have gradually joined the provincial health system, improving cultural and geographical accessibility of health care to Aboriginal communities. Nonetheless, according to official sources, their performance have weaknesses in training and supervision as well as in its coordination with referral hospitals [14]. Health teams have no doctors, biochemists or nurses that belong to the Pilaga or Wichi ethnic groups. Health teams visit indigenous communities according to the number of inhabitants. Indigenous communities with larger population (average 800) have Primary Health Centers or Rural Hospitals with permanent medical care. Communities with an average population of 250 members, are visited by health workers and health teams regularly. In indigenous communities with less than 250 members, the health team visit takes place in schools or community halls. Province of Formosa was divided in twelve health districts. Our study area, located in District II, had programs responsible for immunization coverage, children care, pregnant women and prevention of vector-borne diseases. At the time of our field work, the local health system included both rural and peri-urban primary health care centres and the local referral centers of the National Chagas Program.

The data collected allows for an interpretive description of the Wichi and Pilaga’s semantic and symbolic understandings of Chagas disease and its vector as well as these groups’ relationships with the local health system.

## Methods

Existing links between indigenous community representatives and senior health system staff were key factors in locating the study in peri-urban and rural areas of Las Lomitas, in the centre of Formosa Province (lat. 2º42’ S; long. 60º35’ W). Fieldwork was conducted in 2006-2007. The research design was cross-sectional, qualitative, and comparative. Empirical data came from the testimony of members of the Pilaga and Wichi indigenous ethnicities, obtained through in-depth interviews and participant-observation. The data obtained was analyzed comparatively – by ethnic grouping – to look for intercultural differences that shed light on epidemiological traits of each indigenous group.

The in-depth interviews, conducted and recorded in vernacular language, were carried out by an experienced anthropologist. Shamans, health workers, adults infected with *T. cruzi*, and their relatives from different family groups (15 Pilaga and 14 Wichi) from the 4 communities involved in the project were interviewed (La Bomba, Km 14, Lote 47 and Lote 42). A thematic guide based on culturally-relevant indigenous categories was used [15]. The main topics were: health and disease, traditional prevention methods, medical specialists, shamanic forms of therapy, hospital therapy, Chagas disease, its symptoms, its evolution and treatment, connection to the health system, *vinchuca* bites and their effects.

Data collected in the interviews was recorded with different documentary techniques, transcribed, and archived in databases with software for processing interlinear texts (Shoebox 5.0). The textual information was analyzed taking into account ethnic group and place of residence (peri-urban or rural) of the interviewees. A comprehensive approach was taken in analyzing and categorizing the terminology used by the indigenous populations to explain their behaviours with respect to the disease, including its treatment and control by the health system.

The study was undertaken in accordance with the principles established in the Helsinki Declaration. The protocol was approved by the independent ethics committee of Dr. F.J. Muñiz Hospital, Buenos Aires, Argentina. Interview subjects gave informed verbal consent.

## Results

### Indigenous perceptions of disease and healing

#### Pilaga

The Pilaga know that they are getting sick because they begin to have diminished strength and their body gets weaker^a^:

“[When I am getting sick] I have to be in bed. I feel very weak. I don’t want to walk or do anything..”[J]

In the Pilaga’s worldview, diseases always “enter” the body. A Pilaga man, speaking about his wife, who had a CVA, said that *"bleeding got hold of her”, “it entered her”* through her head, and that’s why her mind is not all there *[C].*

One distinctive characteristic of the Pilaga’s shamanic perspective is that disease is intentionally produced by another person or spirit. The sick person may have some responsibility for the harm being caused to him, as it is in the case of being punished for violating some prohibition imposed by the “owners” or “parents” of the different spots.

In this ethnic group, those responsible for attending to diseases are the shamans or the evangelical healers (also called orators). Shamans are also usually the first to be accused of causing diseases, since they have close links with the diverse spiritual entities capable of producing diseases. However, many people have a spiritual protector who gives them special abilities (e.g., hunting or fishing) and can act on their behalf, when the former expresses the desire to harm another person.

However, there is another figure who also has curative capabilities, the evangelical healer who intervenes with words.

“And I pray for a sick person and a while later it seems you can feel something, as if something had entered him. You lift his hand or touch where he says it hurts. You touch him there, with this spirit that gives you those powers and, I don’t know, they cure the person who is feeling pain.” [O]

The rituals of shamans and evangelical healers can vary and fluctuate with practices that can resemble one type of healer or another.

Definitions of shaman categories in *qoml’aqtak* vary from those used in Spanish, the latter of which are the most common in evangelical Pilaga. In this group, those who use their power to harm and not to cure are referred to as “brujos” (“witches”):

“We call witch (“brujo”) a person who can do harm to another, and healer (“curandero”) a person who can heal them.” [V]

In *qoml’aqtak* shamans can be classified into *pi’oGonaq*, who can both heal and harm by directing spirits, and *qonaGanaGaik*, who only harm, by manipulating objects belonging to the victim (hair, excrements, clothes).

The Pilaga do not rely on a single form of therapy. With the exception of a few who confirm only being treated in a hospital or never going to one, everyone considers it convenient to combine all the therapies at their disposal. The first diagnosis, done by the person themselves or their relatives, defines which type of therapy will be attempted. As usually the disease is diagnosed as harm (“daño”), the shaman is the first choice. In many cases, they indicate to take the patient to the hospital after their intervention to finish healing the body once the disease has been “taken out”. In all cases, no matter which therapy was used first, its failure is followed by attempting the other option.

[How do you decide whether to go to the hospital or to a healer?] “Well, I decide on both ways. For example I trust the witchdoctors. I think that sometimes maybe God gave powers to that person, because the Bible says so. Today there are lots of ways to heal a person. I can also trust doctors. Both methods can cure. That is, I don’t just go one way.” [F]

#### Wichi

Wichi conceptualize illness as a journey (*noyik*) between life and death. The living person (*ilóy*) is transformed into a dead person (*yil*), producing in that process the discharge of the -*hesek* (the soul) and, in many cases, its transformation into a*ahót. Ilóy* and *yil* are two different ways of being and are opposed.

The Wichi concept of illness far surpasses the idea of an individual ailment. Diseases multiply in Wichi villages and one of the roles of the shamans is to confirm the appearance and persistence of these diseases and, if possible, dispel them.

“Every month they [the shamans] get together, for example, if there is a lot of disease. They call everyone to see what is happening with the disease. And they say that the disease is going to go away, or that it’s not going to go away, that it’s going to stay here, for example. And it happens.” [J]

The disease is not only understood from a particular “epidemiological” perspective, but it is also “understood as ‘harm’ in a larger sense as it refers to everything that causes harm to any aspect of human existence” [16]. This is why drought, for example, which affects human life, must also be “cured” by negotiating with the spirits who produce it.

Currently the consensus among Wichi in these communities is that “there are no more *hiyawé* (shamans)” or that “they are all finished” [J]. Nevertheless, at least one person in each extended family group is a traditional or, more frequently, evangelical shamanic healer.

Traditional shamans strongly reject Western Medicine, and tend to sway their people against going to the hospital. On the other hand, there is an increasing acceptance of scientific therapies that are incorporated as complementary therapies, overcoming in many cases the shamans’ fierce opposition.

“And well, now if the prayer doesn’t relieve it, well, we take them to the hospital and they give medicine. (…) That’s the problem when you get sick… sometimes prayer doesn’t cure it. It relieves it, but maybe you need a little medicine, pills. We keep praying for pills that can cure, that can heal. We do that and it seems that God himself answers that prayer and cures.” [F]

For Wichi there is a clear distinction between older diseases, prior to contact with the “white man” and current diseases. In the Wichi’s shamanic perception, diseases or “pains” (*oitáj*) are spiritual beings of *ahót* nature that the shaman can see and manipulate.

The group of older diseases includes: “diarrhea”; “cold”; “cancer”; “measles”; “small pox”; and “broken bones” [P]. Chagas disease is one of the new, unknown diseases. The shaman cannot cure it because based on its origin, it is a sickness that is cured with “medicine”. Reciprocally, there are diseases that Western doctors cannot see, detect, or cure.

“In the hospital you have to examine people. If the person is cursed the doctors don’t know what that person has, because with their equipment they can’t see it. Now when they do know what the illness is, they can make a remedy, and they give it, to cure the illness. If the doctors don’t find anything when they do their studies and their tests, we have to bring the patient to the person who does know.” [J]

“When there is some disease and it is still small it can be cured by the healer. But if it is more advanced, then we bring the person to the hospital. The doctor’s diagnosis tells him that they don’t know what disease the person has. They do all the diagnostic tests, blood, urine, TB test, everything, and if everything comes out negative, they for sure won’t treat the patient, so the patient goes right back to the healer.” [N]

According to the testimonials, people affected by witchcraft, a sickness sent by a shaman (*hiyawé)*, cannot be cured at the hospital*.*

“The witchdoctor knows that what the boy has isn’t natural, that it is the work of another witchdoctor. That’s why their diagnoses [the hospital’s] don’t come out. It is ‘tohē’, it’s not natural. To put it more or less simply, what they call ‘tohē’ is what comes from a witchdoctor.’Tohē’ is something that a really skinny person has, that up to now we can’t fix, and it almost seems that there is no cure for it. It has to be a witchdoctor who can cure it, and sometimes it has to be various witchdoctors working together.” [N]

### Chagas vector and disease: knowledge, beliefs and practices

#### Pilaga

Until 50 years ago, the Pilaga lived in temporary houses, made of branches, where *vinchucas* were not able to make nests. This situation probably led them to consider it an insignificant bug. It does not appear in the ethnic mythology like other animals do:

*“There are* vinchucas *because we live close to the forest in mud huts. Because* vinchucas *live in the forest and fly and look for where the people are. And then they come into the houses and reproduce. That’s when they start to bother people. I have never heard the elders talk about how we are going to cure these bugs. Never… at least I have never heard an elder saying what can we do to combat the* vinchuca*.” [G]*

The worst harm that they attribute to the *vinchuca* is sucking blood, because if the person is bitten by many, they feel weak, which can allow for other diseases to “enter”.

“They are harmful after they bite the sick person a lot and they take all their blood. After comes the flu and then it’s going to end because it has already taken everything. Then more diseases come because the person has very little blood.” [E]

With the exception of the community health workers and with two other indigenous people who had worked as community health workers, those interviewed did not make any connection between the *vinchuca* bite and Chagas disease. According to the testimony of one community health worker:

“Chagas disease is a disease that is transmitted by the vinchuca. Generally it bites when the person is sleeping and based on what doctors say, the person scratches and with this puncture, it spreads the thing they call Trypanosoma cruzi and it enters the blood. The people don’t know that this brings disease. That is, they’re not scared because no one told them that this brings that disease.” [V]

The same community health worker explained what the people in his community say about the effects of the bite:

“Because it isn’t the vinchuca that is sick, but rather it bit a sick person. And it bites us and transmits the disease. Some people say that when the vinchuca bit them it made them very tired. They say adults become very agitated from the vinchuca. They say their heart is working fast, it’s beating fast, and then it goes back to normal.” [V]

The most notable characteristic, from the Pilaga’s perspective, is the silent development of the disease that finally manifests itself suddenly with the death of the individual. In these cases of sudden death, the disease must have been sent by another person.

#### Wichi

Usually the Wichi identify two to three “types” of *vinchucas*. In many cases they say that some are more “poisonous” or that some specialize in humans or animals. These same assertions were made by community health workers who participated in training conferences on Chagas disease.

“They explained to me that vinchucas bite people, that they come out as if they were nocturnal, they come from the forest. And there are different vinchucas, there are vinchucas that aren’t poisonous, there are others that like to bite people. There are others that bite the animals in the forest. There are two vinchucas. There are vinchucas that bite animals and humans. There are the somewhat coloured, the very coloured but very thin that bite the animals. And for people, the other one, the black one.” [X]

*“She [referring to Q, for whom J offered to translate] says that there is a* vinchuca *more dangerous that the one in the houses, and that is the* vinchuca *from the forest. It is striped and small, and it has a smell, she says. There’s another that is yellow, and [according to J] it is the same as the black one once it changes skin.“ [J y Q]*

They also expressed their belief that young *vinchucas* (nymphs) do not transmit the disease.

“The adults, yes. The small ones, no, they don’t have it yet. The adults do, they already have Trypanosoma.” [X]

“When the time comes, they wake up again, they come from the north, in December or January. There are many types, red, which is the one with the most poison, the doctor told me that. When it bites you, a pus forms in the place where it bit you. The one that is more black bites, but they say that only after two years it causes Chagas. The other one that is more red, they say leaves a bump, and it doesn’t cause Chagas. Everyone says it, and I believe it.” [V]

Control of the *vinchuca* is limited by Wichi beliefs. There are some cultural norms that strongly interfere with vector control measures implementation, and in particular spraying. They are, on one hand, the great spatial mobility of the family groups, who develop a circuit of visits to relatives of neighbor communities (some located farther away than 100 km), currently following the rhythm of the evangelic calendar. These visits imply going away from their homes, which are left closed up, several times per year for variable periods of a few weeks. This means that the planned spraying cannot be accomplished at once in the whole community. Neighboring relatives to closed-up houses are not authorized to open them, nor to touch and carry outside personal objects without their owners’ presence. On the other hand, according to Wichi beliefs, the soul of the dead, which becomes *ahót,* tends to return to its home and torment the survivors. Because of this, Wichi have various strategies to confuse the *ahót* so that it cannot find their family. The most common is to destroy their house and build a new one in another place, or, sometimes only a few metres away from the previous one, but changing the location of the doors and windows. Being aware of the problem, a community health worker drew attention to the need to adjust the frequency of insecticide spraying following deaths in the community.

Despite Chagas disease being endemic in the region, for Wichi, who hardly ever mention it, it is something completely novel. In their traditional way of life, they did not build permanent housing, they moved seasonally within a large territory. Their former housing made of branches and meant to last at most a few weeks, met healthier and more hygienic conditions than their current housing.

The majority of the Wichi population is unaware of Chagas disease:

“In the workshops held last year, we asked where do they come from and the doctor told the people that it is a bug that flies. For example when there are sick people here in Formosa and in other places, they fly. And when they get there they bite whoever and that’s it…then we have it.” [J]

There is, however, a consensus that *vinchuca* bites are very unpleasant and can cause weakness.

“They come out when it’s dark, when there’s no light. When lots of them bite you, you wake up feeling weak. The bite is nothing and the weakness lasts one week.” [M]

Many Wichi associate the name of the disease with symptoms related to a general lack of energy, anemia produced by the insect extracting an excessive amount of blood, and with swollen eyes, common in children.

“The person gets skinny, but they don’t die. They get anemic, they don’t have blood. That’s why they have yellow skin and they get white, because the bug sucks lots of blood.” [X]

The difficulty in identifying the “pain” at the beginning and the silent development of the disease are other aspects that differentiate Chagas from “older” diseases.

“The people get sick when it’s already there. Because it’s a very silent disease it progresses, and lots of times, people…let’s say they don’t notify them, they don’t realize. And I don’t even think to go see a doctor.” [E]

When they become aware of the disease, it’s already advanced and the sick person has pain in their heart, they don’t feel like working, they have headaches. Sudden deaths are reinterpreted once the effects of Chagas disease are known.

*“They say that there are some people who die suddenly like that and people don’t know what the disease is and they don’t know if it’s witchcraft, nothing. But the day when the* vinchucas *come out, they thought it was that too. The healthy person, they almost seem like they have nothing, they were walking around, during the day they were walking around and at night the person passes like that, at five in the morning, they pass suddenly. He was telling me that he saw that in many people, that they passed like that and they don’t know what the disease is.” [J]*

### The local health system and aboriginal communities

#### Pilaga

Pilaga accept Western forms of therapy as complementary and at times as a substitute, to their own health framework. Accordingly, they expect community health workers to fulfill certain primary health care tasks such as making routine home visits and distributing medications. The community health worker acknowledges these demands but struggles to meet them because of workload pressures.

“Sometimes there is so much work for us to do as community health workers that we can’t manage. We can’t stay and chat in every house. Because as community health workers we’re always the first to do the work, whether it be weight control in children, monitoring pregnancies, outpatient treatment that doctors prescribe. We give treatments, we give vaccinations and when there is a Chagas program or something we have to spray, when there is dengue we have to spray.” [V]

The social structure that prevails in Pilaga communities affects the interaction between the indigenous population and the community health worker. In some cases, the community health worker cares for large communities, including families with whom they do not have strong kinship ties. The unmet expectations of what the role of a community health worker should be are interpreted by the indigenous population as a consequence of the absence or distance in kinship ties.

In addition to the unmet demands for primary health services, the Pilaga experience problems when they become patients and have to contend with deficiencies in the health system and communication barriers. The former includes: problems of access to prescription medications that can be costly, leading people to look for other therapeutic options [F; G]; and laboratory analyses that are not performed, get lost or need to be repeated, which patients can be reluctant to do [J].

Communication barriers exist in the interactions with doctors and other health system professionals. Doctors prescribe medication without explaining the diagnosis. The lack of explanations can extend to procedures involving surgical interventions [N; V].

Furthermore, Pilaga consider that doctors, like indigenous healers, can “see” diseases. Clinical examinations are seen, from the indigenous perspective, as the quintessential diagnostic technique. For that reason if the tests “come out well” even though the person knows they are sick or if the doctor does not explain what the disease is, the healing process is unsuccessful [Z; B].

“Sometimes when we give in the sample at the hospital, they tell us that it is good, but we are not well. So because of that lots of times I have seen some Aboriginals saying “Why do I go to the hospital if they say my test is good even though I am sick.” And from that point on they begin to mistrust the hospital. One time I resorted to it and they told me that the test says I’m fine. I don’t know what year it was but it must be in my file. But I did the urine, blood, everything. But they told me I was fine, but I was sick.” [F]

While in hospital, indigenous people can be “scolded”, or even mistreated, by nurses or orderlies at the hospital for not meeting the anticipated hygiene level, because their children are playing on the floor or for not immediately “obeying” the nurse’s orders, something that is experienced as discrimination [F; V; J; T].

“And sometimes the nurses look after you well and sometimes they look after you badly. Over there they scold you because since you are Aboriginal, they don’t look after you very well. [What do they say when they scold you?]…You have to…when the nurse calls you have to go urgently.” [L]

There are doctors who speak about Aboriginals with contempt. The testimonies of indigenous people recount, with few exceptions, that doctors do not attend to Aboriginals who do not have an appointment even if they wait until the end of the office hours; while they do this for white people.

#### Wichi

Unlike the Pilaga, among the Wichi groups studied the shamans do not believe in Western therapeutic practices and reject them. Community health workers know that the shamans (*hiyawé*) always advise their people not to go to the hospital. Relationships with doctors are plagued by suspicion and mistrust.

“Because people don’t want to give the bit of blood, for example, that the doctor always asks for. [The hiyawé] tell them for example, that you shouldn’t consult a doctor or you shouldn’t go give blood because it’ll make you feel bad. Sometimes they say that the blood the white people ask for is for them to take away… to sell it, for example. And the people believe it.” [E]

The Wichi health workers are instructed to approach people in the hospital, but they are not always able to do this successfully. Aboriginal health workers can be perceived as privileged for having better salaries and for identifying themselves primarily as hospital employees and secondarily as members of the Wichi community [J]. The divide between the community and the hospital is intensified when they diagnose some serious disease and the medical authorities perform procedures including the mandatory transfer of a patient, carried out with assistance from the police.

“The doctor, when the person arrives asks if the people did the tests and in that instance we have to explain up to three times and then we leave them so that they can talk with the police officer. These days it’s just like that. And the police officers go get the person who doesn’t want to do the test or the person who doesn’t want the lady to take the child to the hospital.” [J]

Doctors are the targets of Wichi criticism, for some of the same reasons expressed by the Pilaga: difficulty in understanding the instructions on how to take medications prescribed to them [J]; tests that are not performed or being informed that “all is well” even when they still feel sick [V]. However, there are other complaints specific to the Wichi, including that doctors prevent Wichi women from attending appointments accompanied by relatives who can act as translators.

“It’s not that people don’t want to go to the doctor. Women are the ones that take care of the family, and some of them don’t understand Spanish. The doctor speaks to them and they don’t understand. For example, my family goes to the doctor and I have to go with them so we can understand a bit.” [V]

During hospitalization, the patients’ companions are mistreated by nurses and orderlies:

“…There are others who don’t want to go because they say that in the hospital the nurses scold the people who are with the patient.” [C]

## Discussion

Adopting a cross-cultural view, both similarities and differences in the Pilaga and the Wichi cultures can be observed. The similarities occur in the ways that both ethnic groups understand illness generally. Diseases are understood as resulting from the violation of the normative order or as a tool that is deliberately used during interpersonal conflicts. Indigenous communities have knowledge systems that formulate generalizations of diseases, classifying them as natural or unnatural or as prior to or subsequent to the appearance of the white population. These classifications have consequences on the way the indigenous population acts. For the Pilaga, the coexistence of shamanic and scientific medicine allows for a greater permeability of health staff in the community. For the Wichi, the opposition between the two concepts of disease generates a greater wariness towards Western medical interventions. There are also similarities in both communities in the acceptance of the role played by the shaman, to whom both groups attribute a particular ability for spiritual mediation and contributions to healing. Nevertheless the presence of Indigenous healers, particularly among the Wichis, has weakened in the last few decades and has developed into a shamanic movement within evangelical cults, supported by a worldview and principles similar to those of traditional shamanism [17].

Cross cultural view also applies when the relationship between indigenous people and health workers and services takes place. In their relations with the local health system, both communities experience situations that jeopardize their access to adequate service provision. Highly relevant on this regard is the fact that the interaction between hospital doctors and indigenous patients is almost completely wordless. It consists of just a few monosyllables and gestures from both parties, allowing the patient to communicate the affected body region. The physician prescribes (sometimes only in writing) the way to obtain the medicine (which is lately given for free in the hospital’s drugstore) and the posology.

In addition, there are hindrances to an adequate service provision related to the prevailing styles of the health professionals or inefficiencies in the system that, as such, can also affect non-indigenous groups. Other situations that weaken the relationship between the indigenous people – of both ethnicities – and the health system include linguistic difficulties and, more generally, the lack of cultural understanding. These results are consistent with the literature. A study conducted in Bolivia in Aymará communities, remarks on the “unproductive communication” that takes place between doctor and patient when the former uses scientific language and the latter recur to colloquialisms in the Andean language [18]. Also, in a research carried out in Argentina, among the Mbya-Guarani communities, it is underlined the lack of ability of the health system to “differentiate according to the culturally specific contexts within which health outreach and access is needed” [7].

Perceiving *vinchuca* as a health risk seems to be at odds with cultural heritage. Neither the Pilaga or the Wichi perceive the *vinchuca* as a health risk, and that has consequences on their attitudes with regards to vector surveillance and control. Due to a lack of information, indigenous people of both ethnicities do not associate it either with the disease, or with sudden death. It is unlikely, therefore, that belief systems that orient indigenous behaviours will encourage the adoption of practices for the prevention of household infestations and consequently Chagas disease.

Vector control actions eventually imply a dysfunction between technical and cultural norms. The current National Chagas Program technical guide instructs that when houses are reported as infested with *vinchuca*, spraying is mandatory [19]. Wichi and Pilaga cultural norms related to the circuit of frequent households visits, and those related to the avoidance of the houses of people who have died, mean people are away from their homes for variable periods. This obstructs the correct vector control of the planned sprayings, making a simultaneous sweep through the entire communal space impossible to accomplish. Currently, only houses whose inhabitants are present are sprayed, and the others are then sprayed only if *vinchuca* presence is reported by its owners.

## Conclusions

Chagas disease is defined as a neglected infectious disease that is fundamentally associated with conditions of poverty [20,21]. In Argentina, surveillance and control efforts have made effective progress in stopping the transmission of Chagas disease within many underprivileged communities [22]. However, these advances have not reached the Indigenous population, similarly disadvantaged, who are living in endemic regions [5,23-25]. Although control local programs make adaptations following the national guides, the National Chagas Program lacks a protocol to specifically address the incidence of the disease among Indigenous peoples.

At the local system level there have been attempts to implement prevention actions specifically tailored to the needs of the Indigenous population. In Las Lomitas, the local system response was to improve the linguistic and communicational exchange between the Indigenous population and the health professionals through the community health workers from the Indigenous communities. This response has been shown to be insufficient. The emphasis on the instrumental aspect of communication – flowing from the health providers towards the Indigenous population – takes for granted that the conditions required for this to occur are met [6]. One condition is that the health threat that Chagas disease represents must obviously be recognized by the at-risk groups (Pilaga and Wichi). This does not occur as even though both groups have rates of infestation and infection that far surpass those prevalent in other groups; their belief systems promote a way of thinking that does not attempt to identify their causes. Another condition is that in order for effective communication to take place, those individuals who take on the role of experts should agree upon the adopted course of action. In the case studied, this was not necessarily true, due to the diversity of therapeutic approaches of both the health personnel and the healers. The third condition is that the at-risk population should have a level of alarm to the extent that the message clearly expresses the nature of the risk. Among the Wichi and Pilaga a reaction of alarm is unlikely due to the naturalization of the presence of the *vinchuca* in indigenous homes and the absence of an explicit connection between the insect and a disease that is largely silent.

The conditions are not met because Western health care providers tend to ignore the beliefs and customs of the target groups, and because the relationships established between the communities and those responsible for health care delivery are conflictive. The noticeable epidemiological gap between the indigenous and the non-indigenous populations has to do not only with indigenous beliefs and practices, but also with discriminatory behaviours that occur within the health system, factors that weaken efforts to prevent Chagas disease. The impact of culture on health outcomes can be seen in the higher prevalence of infestation and infection among Wichi as compared to Pilaga. These differences are specifically linked to ethnicity and, as such, reflect cultural characteristics that favor infestation and transmission of the disease among Wichi and complicate the integration of Western medical notions as well as a fluid interaction with health services. In terms of the vector control, if the importance of the *vinchuca’s* presence and the need to participate in the sprayings were communicated effectively, and these were planned taking into account religious practices, e.g. the evangelic calendar, greater control effectiveness ought to be accomplished.

The problematic relationship between the indigenous people and the health system should not be seen as unchangeable because of its cultural nature. At the onset of the study, those people not connected to the hospital were unaware of Chagas disease. The awareness campaign undertaken about the disease permitted Wichi and Pilaga to associate symptoms they were already aware of with Chagas disease. On the health system’s end efforts to reduce linguistic difficulties during medical consultation can be addressed with the use of a trilingual medical glossary as well as an anatomical lexicon that was developed as part of the first stage of this investigation.

These results warn us about the need to revise the official methods and procedures currently used in Chagas control efforts in indigenous communities. Overcoming cultural divides requires a greater understanding among health professionals of indigenous patients’ culture and the incorporation of more efficient approaches to communication. For example, when communicating preventive measures, a strategy to effectively reach the population may be through the production of messages that take into account factors identified by indigenous people as a source of discomfort and deterioration of their health condition such as loss of blood and extreme weakness.

Information meetings in community centres should be adapted to the typical units of the indigenous people’s own social organization, i.e., extended families. Moreover, the most appropriate methods of communication should include oral communication, if possible face-to-face, in vernacular language, and with consideration of indigenous social protocol. A more open and respectful treatment of indigenous patients might be a key factor to reach greater levels of health equity. Translation by Aislin Ryan.

## Endnote

^**a**^The interview quotes were transcribed in standard Spanish and then translated into English. The interviewees are identified with a letter between square brackets.

## Competing interests

The authors declare that they have no competing interests.

## Authors’ contributions

ADA took part in the methodological design, data collection and analysis, and the discussion and writing of the article; JB took part in the methodological design and the discussion and writing of the article; CT assisted in the design and discussion of results; GD and ILl discussed results and took part in the writing of the article; SSE contributed to the discussion of results and directed the project. All authors read and approved the final manuscript.
